# Therapeutic angiogenesis using stem cell-derived extracellular vesicles: an emerging approach for treatment of ischemic diseases

**DOI:** 10.1186/s13287-019-1276-z

**Published:** 2019-06-03

**Authors:** Xiaowei Bian, Kui Ma, Cuiping Zhang, Xiaobing Fu

**Affiliations:** 1 Tianjin Medical University, No. 22, Qixiangtai Road, Heping District, Tianjin, 300070 People’s Republic of China; 2 Key Laboratory of Tissue Repair and Regeneration of PLA and Beijing Key Research Laboratory of Skin Injury, Repair and Regeneration, Fourth Medical Center of General Hospital of PLA, 100048 Beijing, People’s Republic of China

**Keywords:** Stem cells, Extracellular vesicles, Exosomes, Angiogenesis, Ischemic diseases

## Abstract

Ischemic diseases, which are caused by a reduction of blood supply that results in reduced oxygen transfer and nutrient uptake, are becoming the leading cause of disabilities and deaths. Therapeutic angiogenesis is key for the treatment of these diseases. Stem cells have been used in animal models and clinical trials to treat various ischemic diseases. Recently, the efficacy of stem cell therapy has increasingly been attributed to exocrine functions, particularly extracellular vesicles. Extracellular vesicles are thought to act as intercellular communication vehicles to transport informational molecules including proteins, mRNA, microRNAs, DNA fragments, and lipids. Studies have demonstrated that extracellular vesicles promote angiogenesis in cellular experiments and animal models. Herein, recent reports on the use of extracellular vesicles for therapeutic angiogenesis during ischemic diseases are presented and discussed. We believe that extracellular vesicles-based therapeutics will be an ideal treatment method for patients with ischemic diseases.

## Background

With the development of society and improvement of living standards, ischemic diseases have become a leading cause of disabilities and deaths in humans. Ischemic diseases are characterized by a reduction of blood supply with limited oxygen transfer and nutrient uptake. Thus, angiogenesis and blood supply reconstruction are key for treatment of ischemic diseases. Current clinical treatments primarily involve medical therapy (thrombolytic drugs and vasodilator drugs [1]) and surgery [2]. However, it remains difficult to achieve the purpose of vascular remodeling using either drugs [3] or surgery [4]. Inspired by the fact that the body undergoes natural angiogenesis in response to an insufficient blood supply, scientists have learned to enhance the efficiency of angiogenesis as a treatment strategy. The concept of therapeutic angiogenesis involves introducing an agent to promote the growth of new blood vessels in ischemic tissue. Stem cell therapy is a technology that has shown great prospects for ischemic diseases [5, 6]. Indeed, stem cells have been used in animal models and clinical trials to treat various ischemic diseases. However, as transplantation of stem cells continues to be limited by ethical issues, tumorigenicity, and immune rejection, such therapies are not widely available in the clinic.

Recent studies have found that stem cell supernatants can promote repair of damaged tissue [7, 8]. Accordingly, researchers began to focus on the exocrine function of stem cells. Extracellular vesicles (EVs), which are secreted from cells, have been discovered for more than 30 years. In the past, scientists have thought of EVs as cellular dust. Today, EVs are thought to be carriers of intercellular biological information, as they may contain nucleic acids, lipids, and proteins, thereby playing an indispensable role in cell-to-cell communication [9]. Furthermore, the composition of EVs varies according to their origin, and the information they carry also varies [10]. Biological characteristics and functions of EVs suggest their potential application for cell-free regeneration strategies, which may avoid the disadvantages of current stem cell transplantation techniques.

In particular, recent studies have reported that EVs accelerate angiogenesis in cellular experiments and animal models [10–12]. Here, we first summarize the characteristics and properties of EVs (Table 1) and then discuss the emerging role of stem cell-derived EVs in ischemic diseases, such as chronic wound, ischemic cardiomyopathy, and ischemic stroke. We believe that EV-based therapeutics will be an ideal option for patients who suffer from ischemic diseases.

**Table 1:** The main characteristics of extracellular vesicles

Biological characteristics	Exosomes	Microvesicles	Apoptotic bodies
Generation	MVEs fuse with cell membranes to release ILVs as exosomes into the extracellular space	budding from plasma membrane directly	budding from apoptotic membrane directly
Shape	Cup-shaped	Heterogeneous	Heterogeneous
Size(nm)	50–150	100–1000	1000–5000
Markers	Tetraspanins (CD9/63/81), Alix, TSG101, flollin, clathrin, MHC	Annexin V, selecns, integrins, flollin-2, CD40, metalloproteinases	Annexin V, Histones
Lipids	PtdSer, sphingomyelin cholesterol, ceramide, lysobisphoshadic acid etc.	PtdSer, cholesterol, sphingomyelin etc.	PtdSer etc.
Nucleic acids	mRNA, miRNA, lncRNAs	mRNA, miRNA, lncRNAs	mRNA, miRNA, lncRNAs, fragments of DNA

*Abbreviation*: *MVEs* multi-vesicular endosomes, *ILVs* intraluminal vesicles, *PtdSer* phosphatidylserine

### Biological characteristics of EVs

EVs have three distinct types including exosomes, microvesicles (MVs), and apoptotic bodies (ApoBDs) [13], as classified by their biogenesis and origin. Here, we mainly summarize the generation, composition, and isolation of EVs.

#### Generation of EVs (Fig. 1)

Exosomes, defined as 50–150-nm-sized vesicles, were found and named in 1987 [14]. The process of exosome generation can be summarized into three parts. First, the cytomembrane recesses inward to form early endosomes. Second, these early endosomes further develop into multi-vesicular endosomes (MVEs) in which intraluminal vesicles (ILVs) are formed by intraluminal budding. Finally, MVEs fuse with cell membranes to release ILVs as exosomes into the extracellular space, where they can be taken up by donor cells [9]. Released exosomes can travel to distant tissues to affect the behavior and biological function of target cells [15], which bind to the surface of exosomes through specific ligands. There are two ways in which exosomes enter target cells [16], namely cellular endocytosis and membrane fusion, whereby they release their cargoes. Unlike exosomes, MVs are in the range of 100–1000 nm in diameter [17] and are usually larger than exosomes. MVs bud from plasma membrane directly and then are released extracellularly under the condition of various stresses including irradiation, injury, and hypoxia [18]. Many studies have shown that exosomes and MVs are generated from healthy cells, while ApoBDs are mainly produced by dying cells or apoptotic cells [19]. The role of ApoBDs in intercellular communication is currently unclear. Researchers consider the primary functions of ApoBDs are self-cleaning of aging cells and intercellular immune regulation [20–22].

**Fig. 1:**
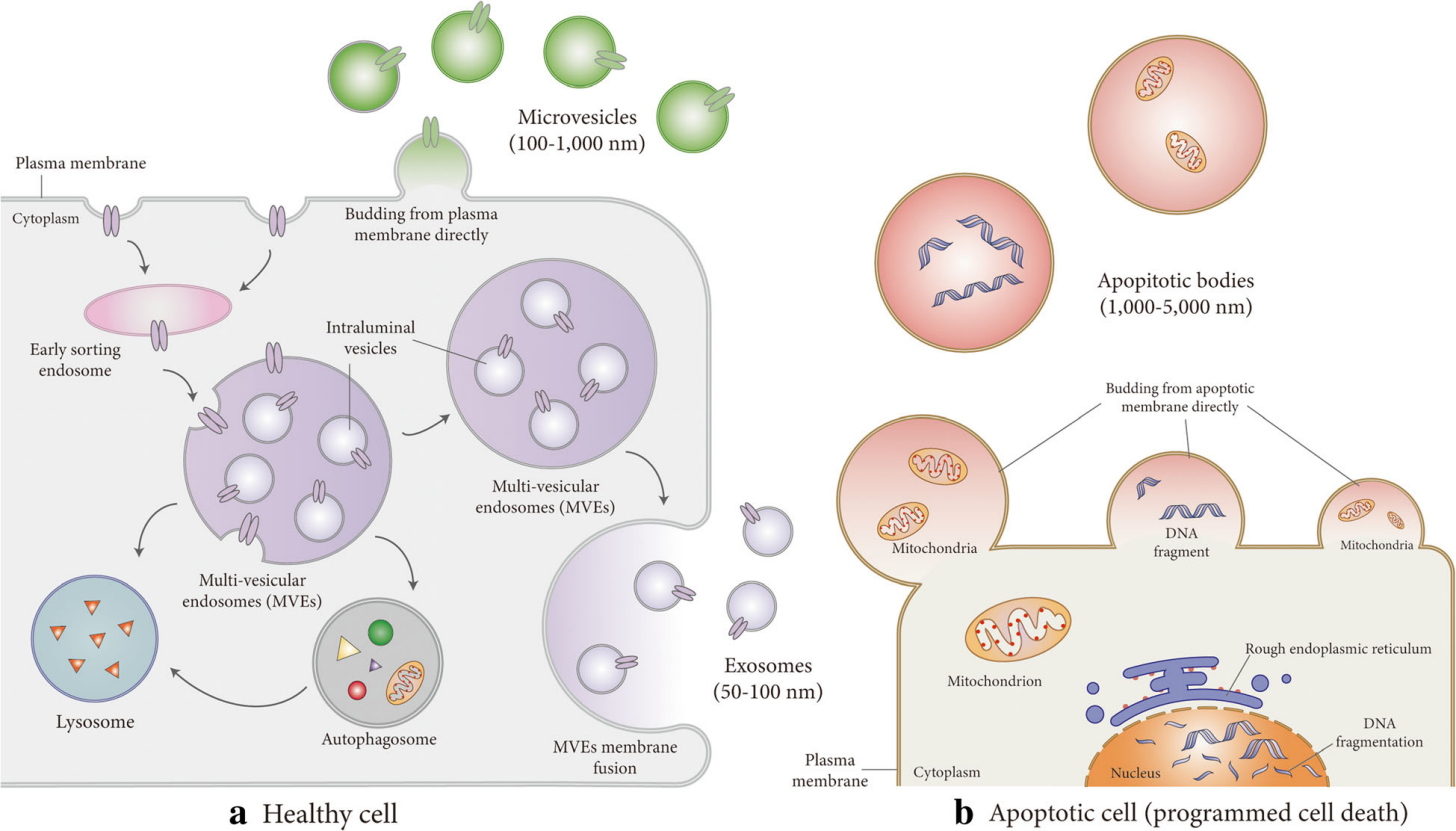
Generation and release of extracellular vesicles (EVs). **a** Healthy cells produce exosomes and MVs. Exosomes occur through three steps: cytomembrane recess inward to form early endosomes, intraluminal vesicle forming in multi-vesicular endosomes (MVEs) by intraluminal budding, and MVE fusing with cell membranes to release ILVs as exosomes. MVs bud outward directly from the plasma membrane. **b** Apoptotic cells produce ApoBDs. ApoBDs bud outward directly from the apoptotic membrane. ApoBDs are thought to be connected with self-cleaning of aging cells and intercellular immune regulation

#### Composition of EVs

##### Proteins

Proteins in EVs are mainly derived from plasma membrane, cytosol, Golgi, and nucleus [23, 24]. As more EV proteins are identified, it has been apparent that EVs contain a common set of EV proteins and cell-type-specific components. The common proteins include cytoskeletal proteins, heat-shock proteins, metabolic enzymes, annexins, ribosomal proteins, tetraspanins, vesicle trafficking-related proteins, and major histocompatibility complex (MHC). The purity of EV preparation is often demonstrated by protein markers enriched in EVs. In fact, tetraspanins including CD9, CD63, CD81, and CD82; heat-shock proteins (e.g., HSP70 and HSP90); MHC classes (I and II); Tsg101; 14-3-3 proteins; and the endosomal sorting complex required for transport (ESCRT-3) binding protein Alix have been regarded as “specific” exosomes markers for years. However, these proteins can also be detectable in ApoBDs and MVs [24, 25]. In addition, the types of cell-type-specific proteins are dependent on their parental cells and conditions under which the EVs are secreted. These proteins include immune-modulating proteins, cell-surface antigens, proteases, angiogenic and molecules [26].

##### Lipids

EVs are rich in lipids such as cholesterol, phosphatidylserine, diglyceride, phospholipid, phosphatidylcholine, phosphatidylinositol, polyglycerol, and phosphatidylethanolamine. Specifically, exosome plasma membranes contain a lot of cholesterol, sphingomyelin, ceramide, lipid rafts, and phosphatidylserine. MV and ApoBD membranes have high concentration of phosphatidylserine [27]. The stability of EV membrane is attributed in part to the lipid content of their membranes [28]. As a result of their high lipid content, EVs have the capacity to pass through biological barriers, escape phagocytosis by the reticuloendothelial system, and protect informational molecules contained within EVs [29]. Interestingly, lipids contained in EVs are somewhat different from other lipids present in their source cells, which might be affected by the micro-environment around EVs. For example, tumor micro-environments may lead to an enrichment of certain tumor progressive or immunosuppressive lipids, such as prostaglandins [30].

##### Nucleic acids

Besides proteins and lipids, EVs also incorporate coding RNA (mRNAs), non-coding RNAs (nc-RNAs), and DNA fragments [31–33]. According to nucleotide length, nc-RNAs are divided into long nc-RNAs (lncRNAs, longer than 200 nucleotides) [34] and small nc-RNAs (sncRNAs, smaller than 200 nucleotides) [35]. LncRNAs encompass the largest proportion of the non-coding transcriptome, but their functions are so far not well defined except for their role in tumor genesis [34]. SncRNAs in EVs include microRNAs (miRNAs), mitochondrial RNAs, piwi-RNA (pi-RNAs), small nuclear RNA, small nucleolar RNA (snoRNA), transfer RNA, Y-RNA, vault RNA, and small interfering RNA (siRNA) [36–38]. Among these sncRNAs, miRNAs [39], Y-RNAs [37, 40], pi-RNA [41], snoRNA [42], and siRNA [43] have been shown to mediate the therapeutic effect of EVs. MiRNAs in particular are the well-known group of sncRNAs and have already been studied extensively. In addition, DNA fragments in EVs such as ApoBDs may be related to cell apoptosis [19].

#### Isolation of EVs

The most common method for EVs isolation is differential centrifugation, which can separate similarly sized vesicle particles. Johnstone et al. originally developed differential centrifugation for the separation of EVs in reticulocyte tissue culture fluid [14]. Later, Théry et al. optimized and improved this method [15]. The first step involves centrifugation at 300×*g*, 2000×*g*, and 10,000×*g* to remove cells, dead cells, and cell debris, respectively. The second step is ultracentrifugation (> 100,000×*g*) to obtain a crude EV-rich extract. The third step repeats ultracentrifugation twice to remove contaminating proteins, which allows clear EVs to be obtained. Today, this method is widely used for various biological samples and considered a “gold standard” for isolating EVs. Advantages of this method are simple operation and production of a large number of EVs. However, the whole process is time-consuming and repeated centrifugation operations may damage the EVs. Thus, further improvement of this method is necessary.

### EVs promote angiogenesis in chronic wound healing

Chronic wounds, which have the characteristics of complex pathogenesis, prolonged disease, easy recurrence, prolonged treatment time, high cost, and high disability rate, refer to wounds that cannot attain anatomical and functional wound healing standards after regular treatment for 4 weeks or more [44]. Most recalcitrant wounds result from pressure ulcers [45], diabetic ulcers [46], venous ulcers [47], vascular insufficiency (e.g., arteriosclerosis [48] or critical limb ischemia [49]), and trauma such as burns [50] and frostbite [51]. Difficulties associated with chronic wound healing have primarily been ascribed to a lack of angiogenesis [52]. Furthermore, without neovascularization, acute wounds will become chronic wounds [53]. Recently, EVs derived from many sources of stem cells have been reported as one of the most promising treatments for chronic wounds by promoting angiogenesis (Table 2).

**Table 2:** Extracellular vesicles derived from stem cells promote angiogenesis in chronic wound healing

EVs source	EVs type	EVs isolation	Experimental model (target cells/animal models)	Functional cargo	Molecules/pathways activated	Key functions/downstream genes	Reference
BM-MSCs	Exosomes	Differential centrifugation Ultracentrifugation	In vitro (HUVECs)	STAT3	Akt, ERK, and STAT3	HGF, IL-6, IGF-1, NGF and SDF1↑	Shabbir et al. [54]
BM-MSCs	Exosomes	Differential centrifugation PEG-US-S purification Ultracentrifugation	In vitro (HUVECs)	Wnt3a	Wnt pathway	CD63+ exosomes are a significant carrier of exterior Wnt3a which results in angiogenesis in vitro.	McBride et al. [55]
ADSCs	MVs	Differential centrifugation 100 KDa molecular filtration Ultracentrifugation	In vitro (HUVECs) In vivo (mice/full-thickness wounds model)	/	PI3K-AKT and ERK signaling pathways	VEGFA, PDGFA, EGF and bFGF↑	Ren et al. [56]
ADSCs	Exosomes	Differential centrifugation 0.2-μm pore membrane filtration Ultracentrifugation	In vitro (HUVECs) In vivo (male BALB/c nude mice)	miR-125a	Speculated to Notch signaling pathways	proangiogenic genes Ang1 and Flk1 ↑ anti-angiogenic genes Vash1, TSP1 and DLL4 ↓	Liang et al. [57]
ADSCs	Exosomes	Differential centrifugation 0.2-μm pore membrane filtration Ultracentrifugation	In vitro (HUVECs) In vivo (nude mice)	/	PKA signaling pathway	proangiogenesis gene Angpt1 and Flk1↑ VEGF↑ anti-angiogenic gene Vash1↓	Xue et al. [58]
UC-MSCs	Exosomes	Differential centrifugation 0.2-μm pore membrane filtration Ultracentrifugation	In vitro (HMECs) In vivo (male C57BL/6 mice/full-thickness excisional skin wounds model)	miR-21-3p	PI3K/Akt and ERK1/2 signaling	PTEN and SPRY1↓	Hu et al. [59]
UC-MSCs	Exosomes	Differential centrifugation 100 kDa molecular weight cut-off (MWCO) hollow fiber membrane Ultracentrifugation	In vitro (EA.hy926 cells) In vivo(rats/deep second-degree burn wounds model)	Wnt4	Wnt pathway	Wnt4 induces β-catenin activation in endothelial cells and exerts proangiogenic effects.	Zhang et al. [60]
PMSCs	Exosomes	Differential centrifugation 0.2-μm pore membrane filtration Ultracentrifugation	In vitro (HMECs) In vivo (nude mice/auricle ischemic injury model)	/	/	PMSC-Exos enhanced angiogenesis in vitro and in vivo	Komaki et al. [61]
iPSCs	Exosomes	MagCapture Exosome Isolation Kit	In vivo (male C57BLKS/J-Leprdb (db/db) mice/full-thickness excisional skin wounds and diabetes model)	/	/	iPSC-Exos significantly increased micro-vessel of full-thickness excisional skin wounds in diabetes mice	Kobayashi et al. [62]
iPSCs	Exosomes	Differential centrifugation 0.2-μm pore membrane filtration Ultracentrifugation	In vitro (HUVECs) In vivo (female Sprague-Dawley rats/full-thickness skin defect model)	/	/	iPSC-Exos can increase proliferation, migration, and tube formation of HUVECs in a dose-dependent manner	Zhang et al. [63]
EPCs	Exosomes	Differential centrifugation 0.2-μm pore membrane filtration Ultracentrifugation	In vitro (HMECs) In vivo(male Sprague-Dawley rats)	/	/	eNOS, IL-8, ANG-1, E-selectin, VEGFA, VEGFR-2, HIF- 1a, CXCL16 and PDGFA↑ PDGFB and MMP-9↓	Li et al. [64]
EPCs	Exosomes	Differential centrifugation 0.2-μm pore membrane filtration Ultracentrifugation	In vitro (HMECs) In vivo (male Sprague-Dawley rats/diabetic model)	/	/	aFGF, eNOS, IL-8, ANG-1, E-selectin, VEGFA, VEGFR-2 and CXCL-16↑ MMP-9↓	Li et al. [65]

*Abbreviation*: *BM-MSCs* bone marrow-mesenchymal stem cells, *ADSCs* adipose-derived stem cells, *UC-MSCs* umbilical cord mesenchymal stem cells, *PMSCs* placenta tissue mesenchymal stem cells, *iPSCs* induced pluripotent stem cells, *EPCs* endothelial progenitor cells, *MVs* microvesicles, *PEG-UC-S* polyethylene glycol-sucrose cushion method, *HUVECs* human umbilical vein endothelial cells, *HMECs* human microvascular endothelial cells, *HGF* hepatocyte growth factor, *IL-6* interleukin-6, *IGF-1* insulin-like growth factor-1, *NGF* nerve growth factor, *SDF1* stromal-derived growth factor-1, *VEGFA* vascular endothelial growth factor A, *PDGFA* platelet-derived growth factor subunit A, *EGF* epidermal growth factor, *bFGF* basic fibroblast growth factor, *DLL4* delta-like 4, *VEGF* vascular endothelial growth factor, *IL-8* interleukin-8, *ANG-1* angiopoietin-1, *VEGFR-2* vascular endothelial growth factor receptor 2, *HIF-1a* hypoxia-inducible factor 1 alpha, *PDGFA* platelet-derived growth factor subunit A, *PDGFB* platelet-derived growth factor subunit B, *MMP-9* matrix metallopeptidase 9

#### BM-MSC-EVs

Bone marrow-mesenchymal stem cells (BM-MSCs) are a type of adult stem cells derived from the mesoderm, which mainly exist in bone marrow stroma to support hematopoiesis. Transplantation of BM-MSCs seeded in a collagen scaffold resulted in increased wound healing and enhanced angiogenesis [66]. Furthermore, when BM-MSCs were seeded directly onto the wound site and injected into the wound edges, increased dermal vascularity was observed in the wound [67]. Some studies have suggested that the paracrine functions of BM-MSCs elicit angiogenesis in the wound by activating vascular endothelial cells [68–70]. EVs as an important paracrine factor of BM-MSCs (BM-MSC-EVs) have been examined as potential BM-MSC-based therapies. Experiments showed that BM-MSC-EVs were internalized by human umbilical vein endothelial cells (HUVECs) and promoted endothelial angiogenesis in vitro [54]. This finding is in line with many reports demonstrating the angiogenic potential of BM-MSC-conditioned medium. Further research demonstrated that BM-MSC-EVs activated important signaling cascades including AKT, STAT3, and ERK in recipient cells. These pathways were probably responsible for increased transcription of vascular endothelial growth factor (VEGF), basic fibroblast growth factor (bFGF), and transforming growth factor beta (TGF-β), which can improve endothelial neovascularization. In another study, CD63^+^ exosomes were isolated by flow cytometry of magnetic beads coated with anti-CD63^+^ antibodies. For the first time, CD63^+^ exosomes containing Wnt3a exteriorly were found to stimulate tube length formation in vitro by activating the canonical Wnt signaling pathway [55].

#### ADSC-EVs

In addition to their self-renewal ability and multi-directional differentiation potential, adipose-derived stem cells (ADSCs) are an abundant resource that can rapidly expand in vitro. Indeed, ADSCs have been shown to maintain both a stable phenotype and multipotent differentiation ability after in vitro culture for 40 generations or cryopreservation [71]. In vivo experiments have revealed that ADSC-derived therapies can significantly improve mean capillary count in chronic wounds [72, 73]. Moreover, ADSC-induced acceleration of VEGF levels in diabetic wounds reportedly regulates local angiogenesis [74]. Recently, Ren et al. [56] demonstrated that ADSC-derived MVs (ADSC-MVs) promoted tube formation of HUVECs seeding in a transwell system. Similarly, ASC-MVs could also increase the establishment efficiency of newly formed vessels and mature vessels in vivo. Moreover, after the treatment of ADSC-MVs, the expression of many growth factors and receptors including platelet-derived growth factor subunit A(PDGFA), vascular endothelial growth factor A(VEGFA), bFGF, hypoxia-inducible factor-1α (HIF-1α), VEGF receptor 2 (VEGFR2), and platelet-derived growth factor receptor (PDGFR) were significantly upregulated in HUVECs. Further study showed that ASC-MVs could increase the activation level of ERK and AKT signaling pathways in HUVECs, which may be responsible for the angiogenesis effect of ASC-MVs on endothelial cells. It was observed that loss of Nrf2/ARE activity increases oxidative stress, which can aggravate endothelial dysfunction and abnormal angiogenesis occurring in diabetes [75]. The researchers demonstrated that ADSC-derived exosomes (ADSC-Exo) overexpressing Nrf2 increased granulation tissue formation, promoted tube formation, and accelerated angiogenesis, suggesting that ADSC-Exo can potentially promote angiogenesis. Therefore, transplantation of exosomes may be suitable for clinical applications to treat diabetic foot ulcers. In addition to this study, other scientists reported that treatment of HUVECs with ADSC-Exo can promote the expression of proangiogenesis genes *Angpt1* and *Flk1* and inhibit the anti-angiogenesis gene *Vash1*, which improved wound healing [57]. *Angpt1* and *Flk1* are key for promoting tube formation, whereas *Vash1* inhibits tube formation. Further study found that, by activating PKA pathway signaling, hypoxia-exposed ADSC-Exo promoted proangiogenesis gene expression, downregulated anti-angiogenic gene expression (*Angpt1* and *Flk1*), and promoted tube formation (*Vash1*). In addition, in vivo experiments assessing vascular formation yielded similar results to in vitro cell models, suggesting that hypoxia-exposed exosomes can indeed enhance angiogenesis [58]. A large number of studies have reported that under conditions of hypoxia and inflammation, most cells can secrete VEGF, which can specifically act on vascular endothelial cells and promote blood vessel formation in vivo. As VEGF is known to activate the PKA signaling pathway [76], researchers examined the PKA signaling pathway in HUVECs after exosome treatment. They found that PKA signaling was activated, which further promoted endogenous VEGF expression in HUVECs and synergistically regulated the expression of downstream proangiogenic genes *Angpt1* and *Flk1*, and decreased the anti-angiogenic gene *Vash1*, thus promoting angiogenesis. Therefore, this finding may represent a novel therapy for hypoxic-condition wounds, as well as the treatment of ischemic diseases with stem cell-derived products.

#### UCB-EVs, UC-MSC-EVs, and PMSC-EVs

Fetal appendage-derived MSCs are obtained from both maternal and fetal origins, such as umbilical cord blood MSCs (UCB-MSCs), umbilical cord MSCs (UC-MSCs), and placenta tissue MSCs (PMSCs). Fetal appendage-derived MSCs are an attractive source of transplantable stem cells for wound repair because they have no risk to donors, easy accessibility, and a low incidence of graft-versus-host disease [77, 78]. Many studies have shown that direct use of fetal-derived MSCs or their conditioned media can significantly increase neovascularization and promote chronic wound healing. MSCs derived from fetal appendages secrete proangiogenic molecules including VEGF, hepatocyte growth factor (HGF), bFGF, TGF-β, and insulin-like growth factor-1 [79–83]. Hu et al. first demonstrated that local transplantation of umbilical cord blood-derived exosomes (UCB-Exo) induced prominent regenerative effects in wound healing, mainly through new blood vessel formation [59]. Further experiments demonstrated that UCB-Exo accelerated cutaneous wound healing through miR-21-3p-mediated promotion of angiogenesis. Zhang et al. later demonstrated that human UC-MSC-Exo improved the tube-formation ability of endothelial cells in vitro and promoted angiogenesis in a cutaneous burn model in vivo [60]. Further exploration of the underlying mechanism revealed that extracted exosomes contained wnt4 protein, which can promote β-catenin nuclear transfer, activate the wnt/β-catenin pathway in skin, and inhibit E-cadherin expression, thus promoting the angiogenesis of skin. Furthermore, exosomes derived from PMSC-conditioned media (PMSC-Exo) also contained angiogenic factors, which enhanced endothelial tube formation. In vivo laser Doppler blood flow analysis showed that PMSC-Exo also enhanced angiogenesis in a murine ischemic injury model [61].

#### iPSC-EVs

Induced pluripotent stem cells (iPSCs) are generated by reprogramming somatic cells into pluripotent stem cells with the characteristics and functions of embryonic stem cells (ESCs). iPSCs are an abundant potential source of autologous or donor-matched cells for therapy and, therefore, have emerged as a promising alternative to ESCs for stem cell transplantation therapy. Scientists found that use of iPSCs or iPSC-derived fibroblasts with three-dimensional structure could improve wound healing [84, 85]. Recently, EVs derived from iPSCs (iPSC-EVs) were also used to treat diabetic wounds in mice [62]. Newly formed vessels and average vessel density in the exosomes derived from iPSCs (iPSC-Exo)-treated group at day 7 were significantly higher, suggesting that iPSC-Exo can improve diabetic chronic wounds by enhancing vessel density and number. In a rat skin full-thickness defect model, exosomes were found to promote wound blood vessel regeneration and maturation. In addition, iPSC-Exo were also reported to promote HUVEC tube formation in vitro [63]. Although some studies have shown that iPSC-Exo has a great effect on wound angiogenesis, underlying mechanisms have not been clearly explained, which provides direction for our future research.

#### EPC-EVs

Endothelial progenitor cells (EPCs) are mainly found in the bone marrow, umbilical cord blood, and peripheral blood. Recent studies indicate that EPCs can promote diabetic wound repair by facilitating neovascularization and the therapeutic effects of EPCs were attributed to a paracrine mechanism [86, 87]. Studies found that EVs derived from EPCs (EPC-EVs) accelerated the healing of diabetic skin wounds by promoting the regeneration of blood vessels. Experiments in vitro showed that EPC-Exo increased the proliferation and migration of vascular endothelial cells and accelerated expression of vascular-related factors such as VEGF and HIF-1α [64]. Experiments in vivo demonstrated that transplantation of EPC-Exo could accelerate skin wound healing in diabetic rats by positively modulating vascular endothelial cell function [65]. Further research showed that Erk1/2 signaling pathway was the critical mediator during the angiogenic responses of endothelial cells induced by EPC-Exo [88]. However, what EPC-Exo components are transferred into vascular endothelial cells remains unclear.

### EVs promote angiogenesis in myocardial ischemia

With advancements in basic and clinical research of cardiovascular disease, the current clinical treatment of myocardial ischemia involves interventions such as percutaneous transluminal coronary angioplasty and coronary artery bypass grafting, but only for patients whose arteries larger than 2 mm in diameter. Patients with arteries less than 2 mm in diameter, certain diffuse coronary artery lesions, history of multiple surgeries, or lack of arteriovenous grafts are unsuitable for such revascularization techniques [89]. Increasingly, acute myocardial infarction (AMI) leads to acute coronary block and reperfusion injury, which can cause acute ischemia and hypoxia in cardiomyocytes. Myocardial necrosis and apoptosis ultimately lead to myocardial remodeling. Therefore, many studies hope stem cells can proliferate and differentiate into new cardiomyocytes to replace damaged myocardial tissue and improve cardiac function after AMI [90, 91]. However, recent studies have shown that survival and differentiation rates of stem cells in transplanted hearts are very low. Therefore, stem cell transplantation may elicit benefits mainly through paracrine effects [92]. At present, different cell-derived EV therapies for AMI are considered to be the most promising method for repairing damaged myocardium and promoting myocardial vessel regeneration (Table 3).

**Table 3:** Extracellular vesicles derived from stem cells promote angiogenesis in myocardial ischemia

EVs source	EVs type	EVs isolation	Experimental model (target cells/animal models)	Functional cargo	Molecules/pathways activated	Key functions/downstream genes	Reference
CDCs	Exosomes	Differential centrifugation Exoquick Exosome Precipitation Solution Ultracentrifugation	In vitro (HMECs) In vivo (male SCID mice)	miR-146a	/	Enhanced angiogenesis and the density of micro-vessels both in vitro and in vivo	Ibrahim et al. [93]
CDCs	Exosomes	Ultracentrifugation Exoquick exosome precipitation solution	In vitro (HUVECs) In vivo (male SCID-beige mice)	/	/	In vitro: stimulate angiogenesis in a HUVEC angiogenesis assay. In vivo: stimulated capillary reorganization.	Lang et al. [94]
CDCs	Exosomes	Differential centrifugation Ultracentrifugation	In vitro (HUVECs)	miR-126, miR-130a, miR-210	/	Speculate: miR-210→EENA3↓→ tube formation↑ miR-130a→GAX and HoxA5↓→ VEGF and VEGFR2↑→tube formation↑ miR-126→VEGF and bFGF↑, Spred-1↓→ tube formation↑	Namazi et al. [95]
CDCs	Exosomes	450 nm pore membrane filtration PEG ultrafiltration Centrifugation	In vivo (female adult Yucatan mini-pigs/MI model)	/	/	decreased acute ischaemia-reperfusion injury, and halt chronic post-MI adverse remodeling in pigs	Gallet et al. [96]
BM-MSCs	Exosomes	ExoQuick-TC reagent Centrifugation	In vitro (HUVECs) In vivo (female Sprague-Dawley rats/MI model)	/	/	Exosomes accounted for the cardioprotection through the formation of new blood vessels.	Teng et al. [97]
BM-MSCs	Exosomes	ExoQuick-TC reagent	In vitro (HUVECs) In vivo (female Sprague-Dawley rats/MI model)	CXCR4	PI3K/Akt signaling pathway	VEGF ↑ Cardiomyocyte survival↑	Kang et al. [98]
BM-MSCs	Exosomes	Differential centrifugation Ultracentrifugation	In vitro (HUVECs/HMECs) In vivo (male C57bl/6 mice)	EMMPRIN	ERK/Akt signaling pathway	EMMPRIN has powerful proangiogenic effects both in vitro and in vivo	Vrijsen et al. [99]
UC-MSCs	Exosomes	Differential centrifugation 100 kDa molecular weight cut-off hollow fiber membrane Ultracentrifugation	In vitro (EA.hy926 cells) In vivo (male Sprague-Dawley rats/MI model)	/	/	protect myocardial cells and accelerate heart repair by angiogenesis after ischemic injury.	Zhao et al. [100]
ADSCs	MVs	Differential centrifugation Ultracentrifugation	In vitro (HUVECs) In vivo (male C57BL/6 J mice and nude mice)	miR-31	/	FIH1↓	Kang et al. [101]
EnMSCs	Exosomes	0.22-μm pore membrane filtration Exosome isolation reagent Centrifugation	In vitro (HUVECs) In vivo (male Sprague-Dawley rats/MI model)	miR-21-5p	PTEN-Akt pathway	PTEN↓ Akt and VEGF↑	Wang et al. [102]
ESCs	Exosomes	Ultracentrifugation	In vitro (HUVECs) In vivo (male C57BL/6 mice/MI model)	/	/	In vitro: increased tube formation; In vivo: decreased infarct size.	Khan et al. [103]
iPSC	MVs	Differential centrifugation Ultracentrifugation	In vitro (CECs) In vivo (C57BL/6 mice/MI model)	/	/	In vitro: EVs impart cytoprotective properties to cardiac cells In vivo: induce superior cardiac repair with regard to LV function and vascularization.	Adamiak et al. [104]
iPSC-Pg iPSC-CM	Exosomes	Ultracentrifugation	In vitro (HUVECs) In vivo (nude mice/MI model)	/	/	EV may promote cell survival, proliferation of resident cardiac cells, and angiogenesis thereby improving left ventricular function.	EI Harane et al. [105]
CD34+ cells	Exosomes	Differential centrifugation Ultracentrifugation	In vitro (HUVECs) In vivo (nude mice)	miR-126, miR-130a	/	In vitro: promote tube formation in HUVECs In vivo: induced the formation of vessel-like endothelial structures in corneal angiogenesis assays.	Sahoo et al. [106]

*Abbreviation*: *CDCs* cardiosphere-derived cells, *BM-MSCs* bone marrow-mesenchymal stem cells, *UC-MSCs* umbilical cord mesenchymal stem cells, *ADSCs* adipose-derived stem cells, *EnMSCs* human endometrium-derived mesenchymal stem cells, *ESCs* embryonic stem cells, *iPSC-Pg* human-induced pluripotent stem cell-derived cardiovascular progenitors, *iPCS-CM* human-induced pluripotent stem cell-derived cardiomyocytes, *MVs* microvesicles, *HMECs* human microvascular endothelial cells, *HUVECs* human umbilical vein endothelial cells, *CECs* murine cardiac endothelial cells, *SCID* severe combined immunodeficient, *MI* myocardial infarction model, *EMMPRIN* extracellular matrix metalloproteinase inducer, *FIH1* hypoxia-inducible factor 1-alpha inhibitor

#### CDC-EVs

Cardiosphere-derived cells (CDCs) are cardiac progenitor cells that can differentiate into the three major cardiac cell types: cardiomyocytes, endothelial cells, and smooth muscle cells [107]. Previous studies demonstrated that CDCs stimulate angiogenesis and functional improvement by indirect mechanisms in the infarcted myocardium [108, 109]. Treating CDCs with exosome biosynthesis inhibitor GW4869 abolished their cardioprotective and regenerative properties. Subsequent studies paid more attention to the role of EVs derived from CDCs (CDC-EVs) and found CDC-EVs have similar therapeutic effects of CDCs in the treatment of myocardial ischemia [93]. When CDC-Exo were injected into the infarct border zone after AMI, the scar was reduced and necrotic myocardium was repaired with neovascularization. This effect of CDC-Exo was confirmed by Gallet et al. who observed a higher number of arterioles in both infarct and border zones of exosomes derived from CDCs (CDC-Exo)-treated pigs [94]. Further study demonstrated that the function of CDC-Exo in neovascularization of ischemic myocardium was related to the high content of miR-146a. Experiments in vitro also showed that CDC-Exo increased HUVEC tube formation and promoted angiogenesis [95]. In addition, the contents of CDC-Exo can be changed under given conditions. For example, exosomes isolated from CDCs cultured under hypoxia were enriched with proangiogenic miRNAs such as miR-126, miR-130a, and miR-210, which increased tube formation of HUVECs [96]. For treatment of ischemic heart disease, CDCs which are derived from myocardial tissue have lower immune responses compared with other stem cells. Furthermore, allogeneic CDC-Exo did not induce significant immune responses after repeated dosing [110].

#### MSC-EVs

As a treatment for ischemia, EVs play an important role as key transporters of paracrine factors during angiogenesis [111]. For example, scientists observed that BM-MSC-EVs can be internalized by endothelial cells and enhanced HUVEC tube formation. Moreover, fluorescence micrographs showed a large number of functional tubes forming in regions surrounding infarction areas. Subsequent in vivo experiments observed increased blood vessel density in hearts injected with BM-MSC-EVs [97]. CXCR4 serves as a major regulator of stem/progenitor cell activities. CXCR4-enriched BM-MSC-Exo activates PI3K/Akt signaling pathway, leading to an increase of VEGF and cardiomyocyte survival under hypoxic conditions [98]. In vitro models, BM-MSC-Exo stimulated endothelial cell migration and vessel formation via ERK/Akt signaling. To determine the angiogenic effect of BM-MSC-Exo in vivo, exosomes were added to the Matrigel plug and then implanted subcutaneously. The results suggested the enhancement of the influx of vascular cells and the blood vessel formation in the Matrigel plug. Analysis of proangiogenic factors revealed the level of extracellular matrix metalloproteinase inducer (EMMPRIN) was high in BM-MSC-Exo. Knockdown of EMMPRIN leads to, both in vitro and in vivo, a diminished proangiogenic effect [99]. The exosomes were isolated from UC-MSC. In vitro, UC-MSC-Exo could promote migration of endothelial cells and tube formation, which might be associated with the increased expression of Bcl-2 family [100]. Kang et al. observed that MVs from ADSCs, especially from endothelial differentiation medium-preconditioned ADSCs, also enhanced angiogenesis both in vitro and in vivo, but the molecular mechanism was different. The level of miR-31 was found to be upregulated in preconditioned ADSCs. Further study showed miR-31 targeted factor-inhibiting hypoxia-inducible factor 1 (FIH1) in vascular endothelial cells to mediate the proangiogenic effect of MVs [101]. Another research assessed therapeutic properties of BM-MSCs, ADSCs, and endometrium-derived mesenchymal stem cells (EnMSCs) in a rat model of AMI and found that EnMSCs supported enhanced microvessel density. Analyses of exosomal microRNAs revealed miR-21 was the potential mediator of EnMSC therapy via the phosphatase and tensin homolog (PTEN)/Akt pathway [102].

#### ESC-EVs

ESCs have the ability to produce exosomes which are capable of instigating cell analogous response in target cells. In order to assess the therapeutic efficacy of ESC-derived exosome (ESC-Exo) in post-infarct myocardium, ESC-Exo were intramyocardially injected in mice at the time of AMI. After 4 weeks, immunohistochemical analysis showed the capillary density was remarkably increased in ESC-Exo transplanted hearts, but the underlying basis for the effect is unknown [103].

#### iPSC-EVs

In recent years, iPSC researches have offered exciting opportunities for tissue restoration. Scientists compared the angiogenesis ability of iPSCs with that of iPSC-EVs in heart failure. The results demonstrated that both iPSCs and iPSC-EVs significantly promoted the migration and tube formation of murine cardiac endothelial cells (CECs). Further experimental analysis of capillary density in vivo was performed in the infarct zone, border zone, and non-ischemic zone of infarcted mouse hearts respectively. IPSC-EV injection resulted in greater number of capillaries in the infarct zone compared with iPSC injection [104]. Another study observed the EVs from cardiovascular progenitor cells derived from iPSCs (iPSC-CPC-EVs) promoted the migration and tube formation of HUVECs. Moreover, iPSC-CPC-EVs could significantly improve chronic heart failure through decreasing left ventricular volumes and increasing left ventricular ejection fraction [105].

#### CD34^+^ cell-EVs

CD34 is selectively expressed on the surface of hematopoietic stem/progenitor cells and gradually weakens or even tends to disappear with the maturation of the cells. After intramyocardial injection, autologous CD34^+^ cells can enhance myocardial perfusion and function of patients with AMI by promoting angiogenesis [112]. Sahoo et al. investigated the mechanism of CD34^+^ cell-induced proangiogenic paracrine effects and found that the exosomes from CD34^+^ cells (CD34^+^-Exo) have the same effects on endothelial cell viability, proliferation and tube formation on Matrigel as CD34^+^ cells have [106]. Further study showed the therapeutic efficacy of CD34^+^ cell treatment could be increased by secretion of sonic hedgehog (Shh) and exosome-mediated delivery of Shh to AMI represents a major mechanism [113].

### EVs promote angiogenesis in stroke

Stroke is a group of diseases characterized by cerebral ischemic and hemorrhagic injury, with ischemic stroke accounting for 60–80% of strokes [114]. Ischemia and hypoxia cause neuronal degeneration and necrosis, leading to irreversible damage in the ischemic core region [115]. Current effective therapies include the use of tissue plasminogen activator thrombolysis and intravascular thrombectomy. However, the time window for application of these treatments is only a few hours [116]. Moreover, most patients suffer from a certain degree of neurological dysfunction even after receiving effective thrombolytic therapy. Because of these limitations, more than 90% of ischemic strokes cannot be treated promptly and effectively. Therefore, how to reduce ischemic injury and promote the recovery of nerve function in ischemic areas has become a research hotspot. In recent years, a deeper understating of EVs has confirmed that the intercellular information exchange process regulated by EVs is widely involved in angiogenic processes of the cerebrovascular system [117] (Table 4).

**Table 4:** Extracellular vesicles derived from stem cells promote angiogenesis in stroke

EVs source	EVs type	EVs isolation	Experimental model (target cells/animal models)	Functional cargo	Molecules/pathways activated	Key functions/downstream genes	Reference
BM-MSCs	Unclear	0.22-μm pore membrane filtration PEG ultrafiltration Centrifugation	In vivo (male C57BL6 mice/MCAO model)	/	/	Formation of new endothelial cells	Doeppner et al. [12]
BM-MSCs	Exosomes	0.2-μm pore membrane filtration Differential centrifugation Ultracentrifugation	In vivo (male Wistar rats/MCAO model)	/	/	Promote angiogenesis after stroke	Xin et al. [118]
ADSCs	Exosomes	Total exosome isolation kit	In vitro (BMECs) In vivo (male Wistar rats/MCAO model)	miR-181b-5p	TRPM7 axis	TRPM7↓→HIF-1α and VEGF↑ TIMP3↓	Yang et al. [119]

*Abbreviation*: *BM-MSCs* bone marrow-mesenchymal stem cells, *ADSCs* adipose-derived stem cells, *MCAO* middle cerebral artery occlusion model, *TRPM7* transient receptor potential melastatin 7, *HIF-1a* hypoxia-inducible factor 1 alpha, *VEGF* vascular endothelial growth factor

MSCs isolated from various tissues can promote angiogenesis not only in wound healing, but also in stoke [120]. In trying to understand the exact molecular mechanism by which different sources of MSCs exert protective roles in ischemic stroke, many studies have investigated the proangiogenesis ability of EVs. BM-MSC-Exos were used to treat middle cerebral artery occlusion of adult male Wistar rats. The results demonstrated that endothelial cell proliferation, compared with the PBS-treated control group, was significantly increased and new capillary network was formed, suggesting that BM-MSC-Exos promote angiogenesis post stroke [12, 118]. Another research also found that ADSC-Exos which contained miRNA-181b-5p could enhance the tube length of brain microvascular endothelial cells (BMECs) after oxygen-glucose deprivation in vitro [119]. Direct targets of miR-181b-5p were further confirmed. Yang et al. found that the mRNA and protein levels of transient receptor potential melastatin 7 (TRPM7) were declined, and meanwhile, HIF-1α and VEGF were upregulated in BMECs after being cultured with 181b-Exos. These researches suggest that exosomes from stem cells may represent a novel therapeutic approach for stroke recovery.

### EVs promote angiogenesis in other ischemic disease (Table 5)

#### Therapeutic effect of EVs in peripheral arterial disease

Peripheral arterial obstructive disease, caused by atherosclerotic occlusion of the leg arteries, is often accompanied by moderate to severe ischemic pain in limbs, which directly affects the quality of life of patients and imposes a huge economic burden on society and families [128]. Many researches have shown that stem cells such as MSCs and EPCs contribute to angiogenesis after hindlimb ischemia and EVs have been emerging as an important paracrine regulator for stem cells to exert positive therapeutic effects. iPSC-derived mesenchymal stem cells (iMSCs) own powerful therapeutic effects through a paracrine mechanism. Hu et al. reported that exosomes derived from iMSCs (iMSCs-Exo) have the ability to promote angiogenesis after transplantation into ischemic limbs of mice [121]. In another study, exosomes were isolated from human PMSCs cultured with a nitric oxide releasing polymer and revealed superior angiogenic effects on hind limb ischemia in a murine model. Further analysis indicated that enhanced VEGF and miR-126 expressions in exosomes were responsible for exosome promoting angiogenic processes [122]. MVs derived from EPC also contained miR-126 and miR-296 which are known to be angiogenetic, suggesting a role of RNAs transferred by MVs in EPC-derived MVs treatment of severe hindlimb ischemia of mice [123]. CD34^+^ stem cells have been demonstrated to improve perfusion and function of the ischemic limb of patients. CD34^+^-Exo can directly transfer miR-126-3p and mimic the angiogenic activity of their parent cells. MiR-126-3p suppresses the expression of SPRED1 and simultaneously regulates the expression of genes which are involved in angiogenic pathways to promote angiogenesis [124]. In addition, administration of BM-MSC-EVs enhanced the formation of new blood vessels in the ischemic limb. The research on mechanisms revealed the enriched presence of miR-210-3p and VEGF protein in BM-MSC-EV and the high levels of VEGFR1 and VEGFR2 in endothelial cells [125]. The miR-210-3p induces expression of several proangiogenic mRNAs (VEGF and VEGFR2) [129]. Therefore, all above researches indicate that angiogenesis-related miRNAs and proteins are the main components in EVs to exert their proangiogenesis function.

**Table 5:** Extracellular vesicles derived from stem cells promote angiogenesis in other ischemic diseases

EVs source	EVs type	EVs isolation	Experimental model (target cells/animal models)	Functional cargo	Molecules/pathways activated	Key functions/downstream genes	Reference
iMSCs	Exosomes	Differential centrifugation 0.22-μm pore membrane filtration 30% sucrose/D2O cushion purification Ultracentrifugation	In vitro (HUVECs) In vivo (mice/hindlimb ischemia model)	/	/	HIF-1α, TGF-β, VEGFA1, VEGFA2, angiogenin, bFGF, KDR, bFGFR, and VEGF↑	Hu et al. [121]
PMSCs	Exosomes	Differential centrifugation Ultracentrifugation	In vitro (HUVECs) In vivo (mice/hindlimb ischemia model)	miR-126, VEGF	PI3K/AKT signaling pathway	miR-126↑→PIK3R2↓ pAKT↑	Du et al. [122]
EPCs	MVs	Ultracentrifugation	In vivo (SCID mice/hindlimb ischemia model)	miR-126, miR-296	/	VEGF↑	Ranghino et al. [123]
CD34+ stem cells	Exosomes	Ultracentrifugation	In vitro (HUVECs) In vivo (immunocompromised BalbC mice/hindlimb ischemia model)	miR-126-3p	/	VEGF, angiogenin1, and MMP-9↑	Mathiyalagan et al. [124]
BM-MSCs	Size is between in exosomes and MVs	Differential centrifugation Ultracentrifugation Density gradient ultracentrifugation 0.45-μm pore membrane filtration	In vivo (female MC57BL/6 mice/hindlimb ischemia model)	miR-210-3p	/	VEGFR1, VEGFR2, and VEGF↑	Gangadaran et al. [125]
ADSCs	Exosomes	ExoQuick-TC reagent	In vivo (male C57BL/6 J mice/skin flap model)	IL-6	phosphorylation of STAT3	Exosomes treatments led to significantly increased flap survival and capillary density compared with I/R on postoperative day 5	Pu et al. [126]
ADSCs	Exosomes	Differential centrifugation 0.22-μm pore membrane filtration Ultracentrifugation	In vitro (HUVECs) In vivo (male Sprague-Dawley rats/skin flap model)	/	/	ADSC-exos can enhance skin flap survival, promote neovascularization	Bai et al. [127]

*Abbreviation*: *iMSCs* human iPSC differentiate into mesenchymal stem cells, *PMSCs* placenta tissue mesenchymal stem cells, *EPCs* endothelial progenitor cells, *BM-MSCs* bone marrow-mesenchymal stem cells, *ADSCs* adipose-derived stem cells, *HUVECs* human umbilical vein endothelial cells, *VEGF* vascular endothelial growth factor, *IL-6* interleukin-6, *HIF-1a* hypoxia-inducible factor 1 alpha, *TGF-β* transforming growth factor beta, *VEGF-A1* vascular endothelial growth factor A1, *VEGF-A2* vascular endothelial growth factor A2, *bFGF* basic fibroblast growth factor, *bFGFR* basic fibroblast growth factor receptor, *VEGF* vascular endothelial growth factor, *MMP-9* matrix metallopeptidase 9

#### Therapeutic effect of EVs in flap graft

Skin flap transplantation is the most widely used treatment in orthopedic surgery and the most effective treatment for ischemic tissue damage. Adequate blood supply is the basis for improving the survival rate of transplanted flaps. Skin flap transplantation has certain limitations in specific clinical applications, as ischemic necrosis occurs at the distal end of the flap [130]. How to safely and effectively improve the survival rate of transplant flaps and ensure their blood supply has always been a difficult problem for burn orthopedics. Therefore, promoting the angiogenesis of flap grafts is key to solving this problem. With a flap ischemia-reperfusion injury (IRI) model, the capability of ADSCs to protect tissue against IRI were examined. Treatment with ADSCs remarkably increased flap survival when compared with the control group and enhanced expression of proangiogenic genes [131]. Further study demonstrated that ADSC-CM and ADSC-Exo increased tube formation after injection into the flaps and interleukin 6 (IL-6) contained in ADSC-Exo stimulated angiogenesis and led to recovery after IRI [126]. A specific micro-environment can be used for in vitro ADSC culture to develop the customized EVs. Compared with ADSC-Exo and control groups, exosomes isolated from ADSC exposed to low concentration of H_2_O_2_ generated more cord-like structures on Matrigel in vitro and increased blood perfusion and microvascular density in the flap in vivo [127]. These results suggest that low H_2_O_2_ micro-environment facilitates the customized exosome development for cell-free therapeutic applications during skin flap transplantation.

## Future directions and potential limitations (Table 6)

**Fig. 2:**
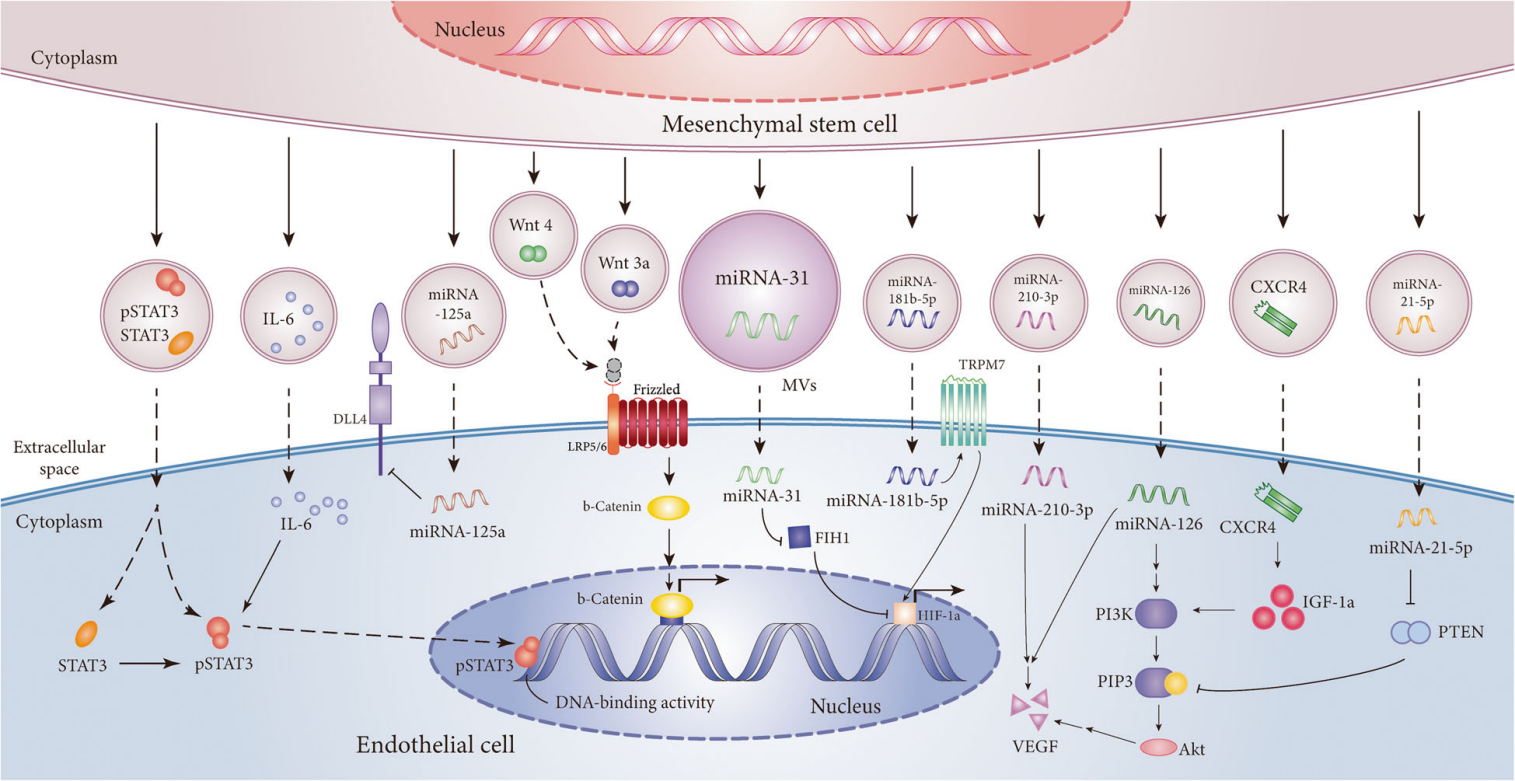
The mechanisms of angiogenesis induced by MSC-derived EVs in ischemic diseases. EVs from BM-MSCs, ADSCs, UC-MSCs, and PMSCs play an important role in neovascularization of ischemic diseases. MSC-derived EVs are enriched with specific cargo molecules including proteins (pSTAT3, IL-6, Wnt 3a, Wnt 4, and CXCR4) and miRNAs (miRNA-31, miRNA-125a, miRNA-181b, miRNA-210, miRNA-126, and miRNA-21). These proteins and miRNAs activate their related signal pathway to regulate the expression of angiogenic factors in endothelial cells. Abbreviation: IL-6, interleukin-6; FIH1, hypoxia-inducible factor 1-alpha inhibitor; HIF-1α, hypoxia-inducible factor-1α; VEGF, vascular endothelial growth factor; PTEN, phosphatase and tensin homolog

**Fig. 3:**
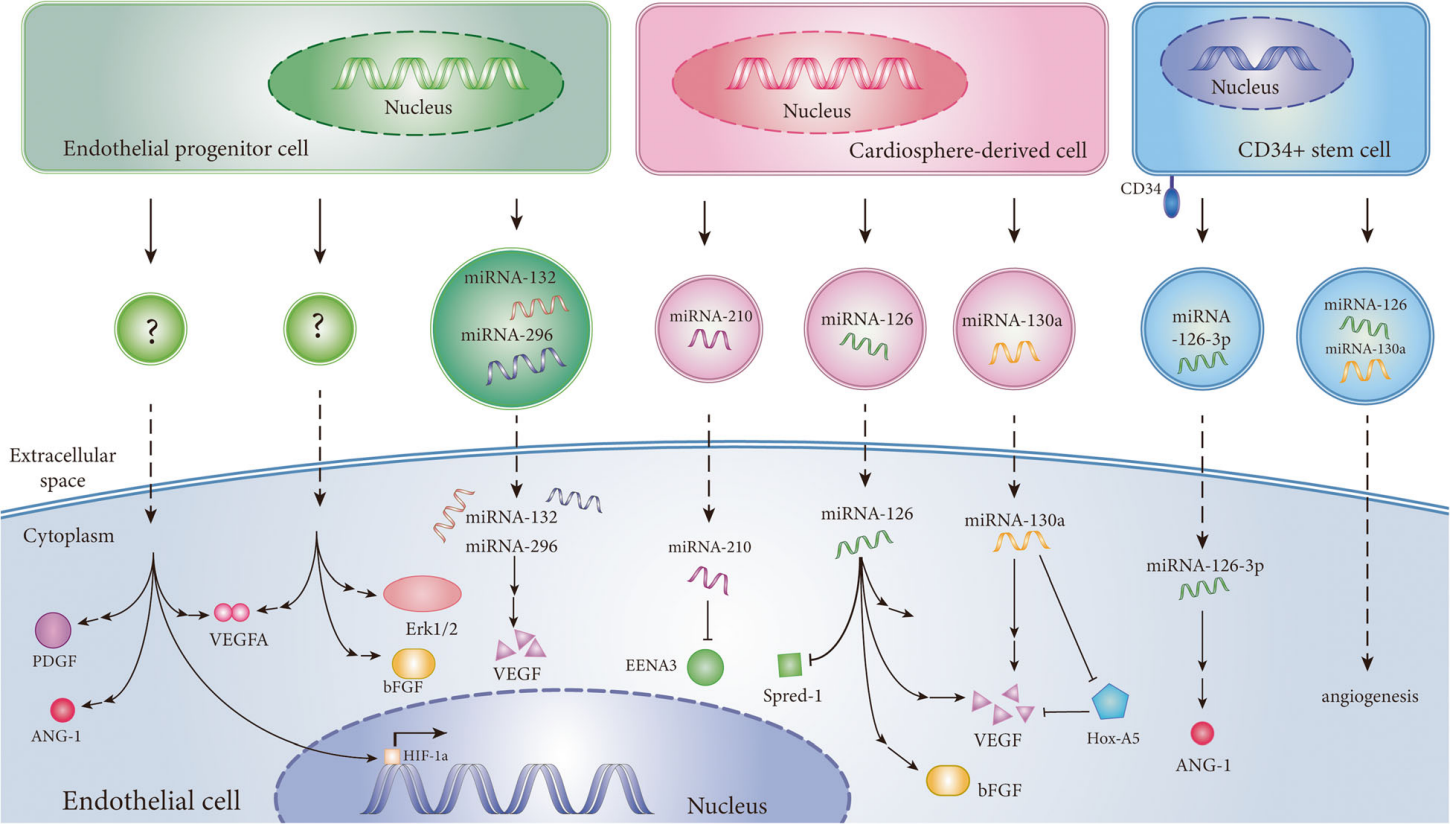
The mechanisms of angiogenesis induced by EVs derived from EPCs, CDCs, and CD34^+^ stem cells in ischemic diseases. EPC-derived EVs promote angiogenesis through upregulating the expression of related transcription factors. CDC-derived EVs are enriched with miR-210, miR-126, and miR-130a, which promote the expression of angiogenic proteins in endothelial cells. EVs derived from CD34^+^ stem cells transfer miR-126 and miR-130 into endothelial cells to stimulate angiogenesis. “?” represents uncertained functional cargo molecules in EVs. Abbreviation: PDGF, platelet-derived growth factor subunit; ANG-1, angiopoietin-1; VEGFA, vascular endothelial growth factor A; VEGF, vascular endothelial growth factor; bFGF, basic fibroblast growth factor; HIF-1α, hypoxia-inducible factor-1α

EVs have opened a new promising avenue for the treatment of ischemic diseases. Angiogenesis-related miRNAs and proteins in EVs derived from MSCs (Fig. 2) and other stem cells (Fig. 3) have shown potential to treat ischemic diseases by directly or indirectly activating angiogenesis-related signaling pathways in target cells. Based on recent researches, many miRNAs including miRNA-21-5p, miRNA-31, miRNA-125a, miRNA-126, miRNA-130a, miRNA-132, miRNA-146a, miRNA-181-5p, miRNA-210, and miRNA-296 are found to promote angiogenesis in ischemic disease [57, 93, 95, 101, 102, 106, 119, 122–125, 135, 139]. VEGF, as a major mediator of angiogenesis, is the most common functional protein component in EVs [125]. In view of the complex components in EVs, other specific functional proteins and miRNAs that play an important role in angiogenesis need to be further identified.

**Table 6:** The advantages and potential limitations of EV therapy

Advantages	Lipid bilayer shell can avert proteolytic degradation; EVs contain many potential regulatory components; EVs can be applied to personalized medicine.	[129, 132–134]
Potential limitations	Short-term effects because of short half-life; Rapid clearance by the innate immune system; Efficiency of EV uptake needs to be improved; Administration routes of EVs must be appropriately selected.	[135–138]

EVs produced by stem cells would be expected to have many advantages in the ischemic environment. First of all, EVs could transfer signals more effectively to target cells because their lipid bilayer shell can avert proteolytic degradation. Scientists are trying to harness the natural ability of EVs to transfer therapeutic payloads into the desired cells. For example, siRNA was effectively delivered by plasma exosomes into the target cells, leading to selective gene silencing of MAPK-1 [132]. Secondly, EVs contain many potential regulatory components such as miRNAs, mRNAs, and proteins. These informational molecules could function simultaneously to generate a strong effect on the characteristics of recipient cells. MiR-210-3p and VEGF protein, as the effective components of BM-MSC-EV, have the same function to promote new blood vessel formation of endothelial cells [125]. Finally, EVs can be applied to personalized medicine [133, 134]. Gene editing in stem cells can produce the desired EVs with specific cell-surface molecules. EVs from gene-edited patient-specific stem cells will hold potential for treatment of ischemic diseases of each individual patient. Furthermore, EVs from iPSC-derivatives can be used for an autologous therapy by activating endogenous repair. We believe that EVs generated from patient-specific iPSC-derivatives probably have a higher angiogenic effect and provide a safer way than stem cell transplantation because EVs used as cell-free therapy are not affected by the ischemic and hypoxic micro-environment and have no tumorigenic risk.

Numerous attempts to treat ischemic diseases with EVs have been made and the results are quite encouraging. However, there are many limitations remaining to be solved. Firstly, EVs transplanted into the damaged tissues may have only short-term effects owing to their short half-life and rapid clearance by the innate immune system. Takahashi et al. showed exosomes from murine melanoma cells disappeared very quickly with a half-time of approximately 2 min from the blood circulation [136]. So how to maintain the retention and stability of EVs over time in vivo is a main challenge in clinical application. Zhang et al. demonstrated that chitosan hydrogel remarkably increased the retention of exosomes in vivo and enhanced the stability of miRNAs and proteins in exosomes, enhancing angiogenesis in ischemic site [135]. Secondly, the efficiency of EV uptake needs to be improved. Cellular uptake of large number of EVs by target cells may improve the effects of angiogenesis. The efficiency of EVs uptake has been found to be related to intracellular and micro-environmental acidity [137]. Finally, the administration routes of EVs must be appropriately selected. Some studies explored whether the angiogenesis effects of EVs are influenced by intravascular injection or local injection of ischemic tissue. Results showed that topical injection of EVs made a better therapeutic effect, while intravascular injection caused EVs to degrade rapidly [138]. In conclusion, we believe that through continued and collaborative efforts, EV-based therapy will yield satisfactory responses in patients with ischemic diseases.

## Abbreviations

ADSC-Exo: Adipose-derived stem cells -derived exosomes
ADSC-MVs: Adipose-derived stem cells derived microvesicles
ADSCs: Adipose-derived stem cells
AMI: Acute myocardial ischemia
ApoBDs: Apoptotic bodies
bFGF: Basic fibroblast growth factor
BMECs: Brain microvascular endothelial cells
BM-MSC-EVs: Bone marrow-mesenchymal stem cells- derived extracellular vesicles
BM-MSCs: Bone marrow-mesenchymal stem cells
CD34^+^-Exo: Exosomes from CD34^+^ cells
CDC-EVs: Extracellular vesicles derived from cardiosphere-derived cells
CDC-Exo: Cardiosphere-derived cells–derived exosomes
CDCs: Cardiosphere-derived cells
CECs: Murine cardiac endothelial cells
EMMPRIN: Extracellular matrix metalloproteinase inducer
EnMSC: Human endometrium-derived mesenchymal stem cells
EPC-EVs: Extracellular vesicles derived from endothelial progenitor cells
EPCs: Endothelial progenitor cells
ESC-Exo: Embryonic stem cell-derived exosomes
ESCRT-3: Endosomal sorting complex required for transport
ESCs: Embryonic stem cells
EVs: Extracellular vesicles
FIH1: Factor-inhibiting hypoxia-inducible factor 1
HIF-1α: Hypoxia-inducible factor-1α
HUVECs: Human umbilical vein endothelial cells
IL-6: Interleukin 6
ILVs: Intraluminal vesicles
iMSCs: Induced pluripotent stem cells-derived mesenchymal stem cells
iMSCs-Exo: Exosomes-derived from induced pluripotent stem cells-derived mesenchymal stem cells
iPSC-EVs: Extracellular vesicles derived from induced pluripotent stem cells
iPSC-Exo: Exosomes derived from induced pluripotent stem cells
iPSCs: Induced pluripotent stem cells
IRI: Ischemia-reperfusion injury
LncRNAs: Long nc-RNAs
MiRNAs: MicroRNAs
MVEs: Multi-vesicular endosomes
MVs: Microvesicles
Nc-RNAs: Non-coding RNAs
PDGFA: Platelet-derived growth factor subunit A
PDGFR: Platelet-derived growth factor receptor
Pi-RNAs: Piwi-RNA
PMSC-Exo: PMSCs derived exosomes
PMSCs: Placenta tissue mesenchymal stem cells
Shh: Sonic hedgehog
SiRNA: Small interfering RNA
SncRNAs: Small nc-RNAs
SnoRNA: Small nucleolar RNA
TGF-β: Transforming growth factor beta
TRPM7: Transient receptor potential melastatin 7
UCB-Exo: Umbilical cord blood-derived exosomes
UCB-MSCs: Umbilical cord blood mesenchymal stem cells
UC-MSCs: Umbilical cord mesenchymal stem cells
VEGF: Vascular endothelial growth factor
VEGFA: Vascular endothelial growth factor A
VEGFR2: Vascular endothelial growth factor receptor 2
